# Personality Traits and Longitudinal Trajectories of Perceived Importance of the Internet Among Middle-Aged and Older Adults in China: A Latent Growth Modeling Study

**DOI:** 10.3390/bs16101735

**Published:** 2026-09-23

**Authors:** Zhu Guo, Ruofeng Xu, Yuqi Miao, Mengyao Li, Yangyi Chen, Yinying Zhang, Maomin Jiang

**Affiliations:** 1 Nursing Department, First Affiliated Hospital of Soochow University, Suzhou 215031, China; 2 School of Nursing, Soochow University, Suzhou 215006, China; 3 Suzhou Medical College, Soochow University, Suzhou 215000, China

**Keywords:** perceived importance of the internet, personality traits, middle-aged and older adults, latent growth model

## Abstract

As digital technologies become increasingly embedded in everyday life, middle-aged and older adults’ perceptions of the importance of the Internet may change over time. However, longitudinal evidence on these changes and their personality-related heterogeneity remains limited. This study examined longitudinal trajectories of perceived Internet importance and their associations with Big Five personality traits among middle-aged and older adults in China. Data were obtained from the 2018, 2020, and 2022 waves of the China Family Panel Studies and included 4790 participants aged ≥ 45 years. Perceived Internet importance was assessed across entertainment, social interaction, learning, and commercial activities. After examining longitudinal measurement invariance, latent growth models were used to estimate initial levels, linear change, and interindividual variability and to examine associations of baseline personality traits with the intercept and slope factors. Perceived Internet importance increased from 2018 to 2022, with significant between-person variation in both initial levels and rates of change. Higher extraversion and openness were associated with higher initial levels but slower subsequent increases, whereas higher neuroticism was associated with a lower initial level but a greater subsequent increase. Conscientiousness and agreeableness were primarily associated with initial levels. After additional adjustment for health and socioeconomic factors, several smaller associations were attenuated, whereas associations involving openness and neuroticism remained comparatively robust. Perceived Internet importance increased over time but followed heterogeneous trajectories. Big Five personality traits showed generally small and differential associations with these trajectories, with comparatively robust associations observed for openness and neuroticism.

## 1. Introduction

Population ageing and the deepening digital transformation are increasingly unfolding in tandem, constituting an important context of contemporary society. As digital technologies become rapidly embedded in information dissemination, everyday consumption, healthcare, social interaction, and public service provision, the internet has become an increasingly important resource through which individuals access information, maintain social connections, and engage with everyday services (Xu et al., 2025; R. Yang et al., 2024). However, the rapid evolution of digital technologies and the continuous expansion of digital application scenarios have placed new demands on middle-aged and older adults in terms of digital adaptation and sustained participation. Some individuals may experience difficulties in maintaining stable and meaningful digital engagement due to factors such as technological updates, changes in physical functioning, and insufficient external support (S. Li et al., 2025; An et al., 2024; Kebede et al., 2022). Against this background, middle-aged and older adults may differ substantially in the extent to which they perceive the internet as important in their daily lives. The perceived importance of the internet reflects individuals’ subjective appraisal of the relevance and value of the internet in everyday life and provides a useful perspective for understanding how they relate to an increasingly digitalized environment. Therefore, understanding how the perceived importance of the internet changes over time among middle-aged and older adults is important for understanding heterogeneity in their engagement with digital environments and informing the development of age-friendly digital services and participation strategies.

The perceived importance of the internet is not necessarily a fixed evaluation that remains unchanged over time. Such perceptions may gradually change as individuals accumulate digital experience and encounter changes in everyday needs, social environments, and opportunities for internet use (Ge et al., 2025). For middle-aged and older adults, whose digital engagement unfolds amid rapidly changing platform rules, technological environments and modes of interaction, the perceived importance of the internet may increase as internet-based activities become more closely integrated into everyday life, while the extent and rate of such change may vary substantially across individuals and life stages (Y. Wang et al., 2024). Existing studies have largely attributed disparities in internet use, digital participation, and related digital outcomes among middle-aged and older adults to structural factors such as educational attainment, income level, urban–rural differences and family support, while paying comparatively limited attention to longitudinal changes in individuals’ subjective evaluations of the internet and the heterogeneity of such trajectories over time (Zhang, 2025; Chen & Wang, 2025; Y. Cui et al., 2024). Although cross-sectional studies are useful for identifying factors associated with the perceived importance of the internet, they cannot how individuals differ in their initial levels or how their rates of change vary over time (Y. Wang et al., 2025). A longitudinal perspective is therefore needed to examine changes in the perceived importance of the internet over time and to identify factors associated with heterogeneous developmental trajectories.

Among the various dimensions of individual differences, personality traits are characterized by their relative stability and explanatory value, providing an important perspective for understanding heterogeneity in the development of the perceived importance of the internet among middle-aged and older adults. Across different contexts, personality traits have been associated with differences in individuals’ cognitive appraisals, behavioral tendencies, and patterns of technology use (Beerbaum et al., 2025; Wu et al., 2024). A meta-analysis has demonstrated systematic associations between Big Five personality traits and information and communication technology use behaviors, suggesting that personality differences may be related to individuals’ tendencies and approaches toward engaging with digital environments (Joshi et al., 2023). The Big Five personality model characterizes individual differences across five dimensions: conscientiousness, extraversion, agreeableness, openness to experience, and neuroticism. Research suggests that individuals with higher levels of extraversion and agreeableness are more inclined to obtain support through social interactions and maintain social connections (Alphenaar et al., 2025; Chao et al., 2024). Individuals with higher openness are more likely to accept changes in social lifestyles and explore new opportunities for participation, whereas conscientiousness may facilitate the development of stable adaptive behaviors (Joshi et al., 2023; An et al., 2024). In contrast, individuals with higher neuroticism may exhibit greater caution and avoidance when facing changes in the digital society due to heightened risk perceptions and experiences of uncertainty (Cheng et al., 2023). Therefore, personality traits may help explain individual differences in how individuals evaluate the importance of the internet and how such evaluations change over time, given their associations with cognitive appraisal, social interaction tendencies, and adaptation to digital environments.

Existing research has mainly linked personality traits to specific aspects of Internet use, including technology adoption, frequency of use, and online participation. Less is known, however, about how these traits are associated with middle-aged and older adults’ subjective evaluations of the importance of the internet, particularly from a longitudinal perspective. In particular, limited evidence is available on whether personality traits are associated with differences in the initial level of the perceived importance of the internet and its subsequent rate of change (Barbosa & de Oliveira, 2026). Therefore, the present study uses data from the 2018, 2020, and 2022 waves of the China Family Panel Studies (CFPS) and applies latent growth models to examine the developmental trajectories of the perceived importance of the internet among middle-aged and older adults. Specifically, this study distinguishes between initial levels and rates of change, examines the associations of personality traits with both dimensions, and conducts age-stratified analyses separately among middle-aged and older adults. By doing so, the study advances understanding of longitudinal changes and individual heterogeneity in how middle-aged and older adults perceive the importance of the internet, while offering insights into how digital services may better correspond to the perceived needs of middle-aged and older adults.

## 2. Materials and Methods

### 2.1. Participants

The data used in this study were drawn from the China Family Panel Studies (CFPS), a nationally representative longitudinal survey designed and implemented by the Institute of Social Science Survey (ISSS) at Peking University. The CFPS tracks changes in China’s social, economic, demographic, educational, and health-related conditions (Xie & Lu, 2015). Launched in 2010, the survey uses a multistage, stratified probability sampling design and is conducted biennially across 25 provinces, autonomous regions, and municipalities in China, with a target sample of approximately 16,000 households. As survey waves after 2022 had not yet been released, this study used data from the 2018, 2020, and 2022 waves.

The 2018 wave included measures of demographic information and personality traits, which allowed the identification of baseline demographic characteristics and personality profiles. The perceived importance of the internet was measured in 2018, 2020, and 2022, providing repeated observations across three time points and allowing its longitudinal trajectory to be examined within a latent growth modelling framework. The three waves were matched using respondents’ unique identifiers. Among 16,944 respondents aged ≥ 45 years in 2018, 9786 remained after matching with the 2020 wave, with 7158 lost to follow-up. After matching with the 2022 wave, 6651 participants were retained, while 3135 were excluded due to unavailable follow-up data. An additional 1861 participants were excluded because of incomplete analytical information, including non-structural missing PII responses, missing personality data, invalid responses, or inconsistent identifiers. The final analytic sample included 4790 participants (Figure 1). Baseline age, sex, and education level were compared between participants included in the final analytic sample and those excluded, and no statistically significant differences were observed in these characteristics (all *p* > 0.05).

### 2.2. Measures

#### 2.2.1. Demographic Information

Gender was categorised (1 = male, 0 = female). Age groups were defined according to participants’ age at baseline in 2018 (1 = middle-aged group, 45–59 years, 2 = older-adult group, ≥60 years); location (1 = rural, 0 = urban), marital status (1 = have a partner, 0 = no partner) and education (0 = illiterate/semi-literate, 1 = primary school, 2 = junior high school, 3 = high school/secondary vocational school, 4 = college and above) were selected as demographic general information for this study.

#### 2.2.2. Big Five Personality Traits

Personality traits are generally regarded as relatively stable and capable of reflecting individuals’ long-term psychological and behavioral tendencies. Therefore, this study used the personality measures collected in 2018 as baseline personality variables. Personality traits among middle-aged and older adults were assessed using the short form of the Big Five Personality Inventory (Wu et al., 2024; Hahn et al., 2012). The dimensions were assessed as follows: conscientiousness (“I do a thorough job,” “I tend to be lazy,” “I do things efficiently”), extraversion (“I am talkative,” “I am outgoing and sociable,” “I am reserved”), agreeableness (“I am sometimes rude to others,” “I have a forgiving nature,” “I am considerate and kind to almost everyone”), openness (“I am original and come up with new ideas,” “I value artistic and aesthetic experiences,” “I have an active imagination”), and neuroticism (“I worry a lot,” “I get nervous easily,” “I am relaxed and handle stress well”). The scale includes 15 items in total, all rated on a five-point Likert scale ranging from 1 = strongly disagree to 5 = strongly agree. Negatively worded items were reverse-coded. Scores for each dimension ranged from 3 to 15, with higher scores indicating higher levels of the corresponding personality trait. The Cronbach’s alpha coefficients for the five dimensions were 0.721, 0.766, 0.795, 0.793, and 0.799, respectively.

#### 2.2.3. The Perceived Importance of the Internet

The perceived importance of the internet (PII) refers to individuals’ subjective evaluation of how important the internet is in different domains of their daily lives. In this study, PII was measured using four conceptually corresponding domains across the 2018, 2020, and 2022 waves: leisure and entertainment, social interaction, learning/information acquisition, and commercial activities. Each item was rated on a five-point Likert scale ranging from 1 = very unimportant to 5 = very important. The four items were summed to generate a composite score ranging from 4 to 20, with higher scores indicating greater perceived importance of the internet. Composite scores were used for descriptive and correlation analyses, whereas the four items at each wave were modeled as indicators of a latent PII factor in the latent growth model (LGM). Before conducting the LGM, longitudinal measurement invariance was examined across configural, metric, and scalar models. Longitudinal measurement invariance was supported across the three levels. The configural model showed acceptable fit (CFI = 0.944, RMSEA = 0.075), with minimal changes after constraining factor loadings (ΔCFI = 0.004, ΔRMSEA = 0.002) and intercepts (ΔCFI = 0.001, ΔRMSEA = 0.001).

### 2.3. Statistical Analysis

Statistical analyses were performed using SPSS version 27.0 and Mplus version 8.3. Descriptive analyses were first conducted to characterize the distributions of the study variables, followed by tests of longitudinal measurement invariance of perceived importance of the Internet (PII) across the three measurement occasions in 2018, 2020, and 2022. An unconditional linear latent growth model was then estimated. The intercept factor represented the initial level of PII in 2018, whereas the slope factor loadings were fixed at 0, 1, and 2 for the 2018, 2020, and 2022 waves, respectively; thus, the slope represented the average change in PII per two-year survey interval. A conditional latent growth model was subsequently fitted, with the Big Five personality traits measured in 2018 included as time-invariant predictors. Baseline age, urban–rural residence, and educational attainment were included as covariates. The primary analyses were performed using robust maximum likelihood estimation (MLR). Unstandardized estimates were reported for the means, variances, and covariance of the growth factors, whereas standardized regression coefficients (*β*) and 95% confidence intervals were reported for the paths from personality traits and covariates to the intercept and slope factors. Model fit was evaluated using CFI, TLI, RMSEA with its 95% CI, and SRMR. Given that the measurement items were rated on five-point ordinal scales, sensitivity analyses were conducted using weighted least squares mean- and variance-adjusted estimation (WLSMV) to assess the robustness of the main findings to the choice of estimator. Additional analyses further adjusted for health status and income level in the original model. Age-stratified analyses were also conducted by fitting latent growth models separately for middle-aged and older adults to characterize the associations of personality traits with the initial level and longitudinal change in PII within each age group (Jiang et al., 2026; Hu & Bentler, 1999).

## 3. Results

### 3.1. Descriptive Statistics

The demographic characteristics of the participants and the results of the univariate analysis are presented in Table 1. The sample consisted of 4790 individuals, with 2502 males (52.23%) and 2288 females (47.77%). The age distribution was as follows: 3591 were in the middle-aged group, aged 45–59 years (74.97%), and 1199 were in the older-adult group, aged 60 years and above (25.03%). The sample included 3176 rural residents (66.30%) and 1614 urban residents (33.70%). Regarding marital status, 339 participants (7.08%) had no partner, whereas 4451 (92.92%) had a partner. In terms of education, 846 participants (17.66%) were illiterate or semi-literate, 1155 (24.11%) had a primary school education, 1607 (33.55%) completed junior high school, 851 (17.77%) had a senior high school, technical secondary school, technical school, or vocational high school education, and 331 (6.91%) held a college degree or higher. Univariate analysis showed that PII differed significantly across age groups, places of residence, and education levels in 2018, 2020, and 2022 (all *p* < 0.001). Significant gender differences were observed in 2020 and 2022 (both *p* < 0.05), whereas the difference was not significant in 2018. No statistically significant differences were found across partnership-status groups in any of the three years (all *p* > 0.05).

### 3.2. Correlation Analysis

Table 2 presents the correlation results of the study variables. Notably, conscientiousness, extraversion, agreeableness, and openness were significantly and positively correlated with PII in 2018, 2020, and 2022 (*p* < 0.001), with correlation coefficients ranging from 0.050 to 0.150. Neuroticism was significantly and negatively correlated with PII at all three time points (*p* < 0.001), with correlation coefficients ranging from −0.104 to −0.036. Additionally, PII was significantly and positively correlated across different time points (*p* < 0.001), with correlation coefficients ranging from 0.284 to 0.474, indicating a degree of temporal stability in PII.

### 3.3. Longitudinal Changes in PII Among Middle-Aged and Older Adults

To examine the longitudinal change in PII from 2018 to 2022, an unconditional latent growth model was constructed, and the detailed results are presented in Table 3. The results indicated a favorable model fit, with χ^2^ = 763.171, df = 61, *CFI* = 0.944, *TLI* = 0.937, *RMSEA* = 0.078, and *SRMR* = 0.074. The mean value of the intercept factor for PII was 7.708 (*SE* = 0.036, *p* < 0.001), and the mean value of the slope factor was 1.348 (*SE* = 0.047, *p* < 0.001), indicating that PII increased significantly over the study period. Meanwhile, there were significant inter-individual differences in the initial level and rate of change in PII. Specifically, the variance of the intercept factor was 1.186 (*SE* = 0.047, *p* < 0.001), and the variance of the slope factor was 0.282 (*SE* = 0.017, *p* < 0.001). In addition, the intercept factor was significantly and negatively associated with the slope factor (covariance = −0.468, *SE* = 0.025, *p* < 0.001), suggesting that individuals with higher initial levels of PII tended to exhibit smaller subsequent increases over time.

### 3.4. Associations of Personality Traits with the Initial Level and Rate of Change in PII Among Middle-Aged and Older Adults

Conditional latent growth models were constructed to examine the associations of personality traits with initial level and rate of change in PII in the overall sample of middle-aged and older adults (*N* = 4790), the middle-aged subgroup (*N* = 3591), and the older-adult subgroup (*N* = 1199). All three models demonstrated acceptable model fit. For the overall sample, the fit indices were χ^2^ = 1015.946, df = 141, *CFI* = 0.918, *TLI* = 0.907, *RMSEA* = 0.069, and *SRMR* = 0.073. For the middle-aged subgroup, the corresponding indices were χ^2^ = 920.187, df = 141, *CFI* = 0.917, *TLI* = 0.905, *RMSEA* = 0.069, and *SRMR* = 0.079. For the older-adult subgroup, the indices were χ^2^ = 783.216, df = 141, *CFI* = 0.928, *TLI* = 0.918, *RMSEA* = 0.067, and *SRMR* = 0.068.

Table 4 and Figure 2 present the associations between personality traits and the initial level and rate of change in PII in the overall sample. Conscientiousness was significantly and positively associated with the initial level of PII (*β* = 0.050, *p* = 0.001) but was not significantly associated with its rate of change. Extraversion was positively associated with the initial level of PII (*β* = 0.146, *p* < 0.001) but was negatively associated with its rate of change (*β* = −0.040, *p* = 0.019). Agreeableness was also significantly and positively associated with the initial level of PII (*β* = 0.055, *p* < 0.001), but it was not significantly associated with the rate of change, with higher agreeableness associated with a higher initial level of PII. Openness was positively associated with the initial level of PII (*β* = 0.139, *p* < 0.001) and was negatively associated with its rate of change (*β* = −0.048, *p* = 0.005). In contrast, neuroticism was significantly and negatively associated with the initial level of PII (*β* = −0.102, *p* < 0.001) but was positively associated with its rate of change (*β* = 0.086, *p* < 0.001).

Table 5 and Figure 3 present the results for the middle-aged subgroup, in which the associations between personality traits and PII trajectories were broadly consistent with those observed in the overall sample, with only minor fluctuations in the estimated coefficients. Table 6 and Figure 4 present the results for the older-adult subgroup. Among older adults, the associations between personality traits and the initial level of PII were generally consistent across personality dimensions, whereas only neuroticism was significantly associated with the rate of change in PII (*β* = 0.108, *p* = 0.003). Conscientiousness, extraversion, agreeableness, and openness were not significantly associated with the rate of change in PII.

### 3.5. Sensitivity Analysis

To examine whether the associations between personality traits and PII trajectories were affected by additional health and socioeconomic factors, a sensitivity analysis was conducted by re-estimating the latent growth model with baseline self-rated health and subjective income status additionally included as covariates, along with age and education. The results were generally consistent with the primary model (Table 7). Specifically, the associations of openness and neuroticism with PII trajectories remained significant with similar effect directions and magnitudes, whereas some associations involving other personality traits were attenuated after additional adjustment. These findings suggest that the main conclusions were generally robust after further adjustment for health and socioeconomic factors.

## 4. Discussion

This study found that PII among middle-aged and older adults increased significantly from 2018 to 2022, with substantial individual differences in both initial levels and rates of change. Personality traits were differentially associated with these growth components: extraversion and openness were associated with higher initial PII and slower subsequent increases, whereas neuroticism was associated with a lower initial level and a greater rate of increase; conscientiousness and agreeableness were associated primarily with the initial level. Age-stratified analyses further characterized these associations within each subgroup, with extraversion, openness, and neuroticism associated with PII change among middle-aged adults, whereas among older adults, personality traits were mainly associated with the initial level and only neuroticism was significantly associated with the rate of change.

### 4.1. Longitudinal Changes in PII Among Middle-Aged and Older Adults

The present study found a significant increase in perceived importance of the Internet (PII) among middle-aged and older adults from 2018 to 2022. During this period, the Internet became increasingly integrated into everyday activities, including social interaction, information acquisition, consumption, healthcare, and public services, particularly with the expansion of online activities and digital services during the COVID-19 pandemic (Seifert et al., 2021; Haase et al., 2021; Lythreatis et al., 2022). The growing integration of digital services into daily life increased the practical relevance of the Internet and may have contributed to higher evaluations of its importance. Thus, the longitudinal increase in PII was consistent with the increasingly prominent role of the Internet in the daily lives of middle-aged and older adults.

Significant variances in both the intercept and slope indicated substantial individual differences in the initial level and longitudinal change in PII. Such differences may be related to prior Internet-use experience, perceived usefulness, and the extent to which Internet functions correspond to individuals’ practical needs (Fang et al., 2024). The negative intercept–slope covariance further indicated that individuals with higher initial PII tended to show smaller subsequent increases. Higher baseline evaluations may leave relatively less scope for further increase, whereas individuals with lower initial levels may exhibit greater increases as Internet-based services become more closely integrated with everyday activities. Ceiling effects and regression to the mean may also contribute to this pattern.

Accordingly, digital services should be more closely aligned with the practical needs of middle-aged and older adults. Improving the relevance of service content, simplifying use procedures, and providing appropriate support may enhance the perceived practical value of digital services, particularly among individuals with lower initial PII.

### 4.2. Associations of Personality Traits with the Initial Level and Rate of Change in PII

Different personality traits were associated with distinct initial levels and rates of change in PII among middle-aged and older adults. Extraversion and openness were associated with higher initial PII but slower subsequent increases. Person–environment fit theory proposes that individuals’ evaluations of their environment depend on the degree of correspondence between personal characteristics and the functions provided by that environment (Lawton & Nahemow, 1973). Previous studies have linked extraversion to social engagement and openness to greater receptiveness to novel information environments and digital technologies (Jin & Qu, 2025; Zhou et al., 2025; Mao et al., 2024). In the context of an increasingly digitalized society, the social, informational, and service functions of the Internet may therefore have shown a relatively high degree of fit with the needs of individuals higher in extraversion and openness at baseline, corresponding to higher initial PII. As Internet use became more widespread and increasingly embedded in everyday life, this early fit advantage may have become more stable, resulting in comparatively smaller subsequent increases.

Neuroticism showed a different pattern, with a lower initial level of PII but a greater subsequent increase. Previous studies have linked neuroticism to greater uncertainty and discomfort in unfamiliar digital environments (T. Wang et al., 2025; Liu et al., 2024), which may correspond to a lower initial fit between Internet-based services and individual needs. Between 2018 and 2022, however, the Internet became increasingly integrated into social interaction, information acquisition, healthcare, consumption, and public services, gradually shifting from an optional digital tool to an important means of meeting everyday needs (S. Cui et al., 2025; H. J. Yang et al., 2025). These changes in the digital environment may have increased the fit between Internet functions and the daily needs of individuals whose initial fit was relatively low, corresponding to the greater increase in PII observed among those with higher neuroticism.

Conscientiousness and agreeableness were positively associated only with the initial level of PII, and both effect sizes were relatively small. Previous studies have linked conscientiousness to goal-directed and planned technology use, and agreeableness to socially supported digital engagement and interpersonal interaction (Wong et al., 2024; Liao et al., 2025; Cohen Elimelech et al., 2025). These characteristics may facilitate an earlier recognition of the practical value of the Internet for everyday tasks, information access, and social connection. As Internet-based services became more widely available and increasingly integrated into routine life, the initial differences associated with conscientiousness and agreeableness were not accompanied by corresponding differences in the rate of change in PII.

From a practical perspective, Internet-based services for middle-aged and older adults should strengthen the fit between digital functions and everyday needs while providing more targeted content and support. Simplifying operational procedures, enhancing social and daily-life service functions, and providing additional support for individuals with lower initial PII may improve the usability and practical value of Internet-based services.

### 4.3. Age-Stratified Associations of Personality Traits with the Initial Level and Rate of Change in PII

Age-stratified analyses further characterized the associations between personality traits and PII trajectories within the middle-aged and older-adult subgroups. Among middle-aged adults, extraversion and openness were associated with higher initial PII and slower subsequent increases, whereas neuroticism was associated with a lower initial level and a greater subsequent increase. Middle age is typically characterized by substantial occupational, family, and social responsibilities, while the Internet has become increasingly embedded in workplace communication, administrative services, household management, mobile payment, and social interaction (Y. Wang & Chen, 2024; He et al., 2022). Within this life context, social engagement, receptiveness to digital services, and adaptation to unfamiliar online environments may be associated with how individuals evaluate the importance of the Internet in daily life (Z. Li et al., 2025; Cheng et al., 2023).

Among older adults, personality traits were primarily associated with the initial level of PII, while neuroticism was also positively associated with the rate of change. Internet use in later life is closely related to functional capacity, everyday needs, and access to supportive resources (Xu et al., 2025; Charness & Boot, 2022). As digital services become increasingly integrated into health management, social interaction, and daily activities, their practical value may become more salient, corresponding to greater subsequent increases among individuals with lower initial perceptions of Internet importance (Z. Yang et al., 2025).

Accordingly, Internet-based services for middle-aged and older adults should be better aligned with their life contexts and practical needs. Strengthening service functions in everyday settings, simplifying operational procedures, and providing appropriate guidance and support may improve the accessibility and perceived value of digital services.

## 5. Limitations

This study has several limitations. First, the CFPS items assessing the perceived importance of the Internet differed to some extent across waves in wording, eligibility, and questionnaire routing. Although longitudinal measurement invariance was examined to evaluate statistical comparability across waves, differences in item content and routing procedures may still affect cross-wave comparisons. Second, personality traits were measured only at baseline, precluding examination of longitudinal changes in personality and their possible dynamic relationships with PII. Third, only three measurement occasions were available, allowing primarily the estimation of linear change and limiting the identification of more complex nonlinear trajectories. In addition, the study period overlapped with the COVID-19 pandemic, and the observed changes in PII may therefore reflect both individual change and concurrent changes in the broader digital environment. Fourth, the use of complete-case analysis, together with longitudinal attrition and non-structural missing data, may have introduced selection bias; accordingly, the findings are most appropriately interpreted with respect to the final analytic sample. In addition, older adults were treated as a single group (aged ≥ 60 years), which may not fully capture heterogeneity across different later-life stages. Fifth, the latent growth models did not incorporate CFPS longitudinal sampling weights, clustering, or stratification, and the estimates should therefore not be interpreted as strict national population estimates. Finally, potentially relevant factors such as household digital resources, prior Internet-use experience, digital skills, technology anxiety, and social support were not fully accounted for. Future studies with additional measurement occasions and richer indicators of individual and digital-environmental characteristics are needed to further examine the long-term trajectory and correlates of PII.

## 6. Conclusions

This study provides longitudinal evidence on changes in the perceived importance of the Internet and their associations with personality traits among middle-aged and older adults. PII increased over time and showed substantial individual variation, while different personality traits were associated with distinct initial levels and patterns of change. These findings suggest that understanding individual differences in perceived Internet importance may contribute to the design of digital services that better correspond to the practical needs of middle-aged and older adults.

## Figures and Tables

**Figure 1:**
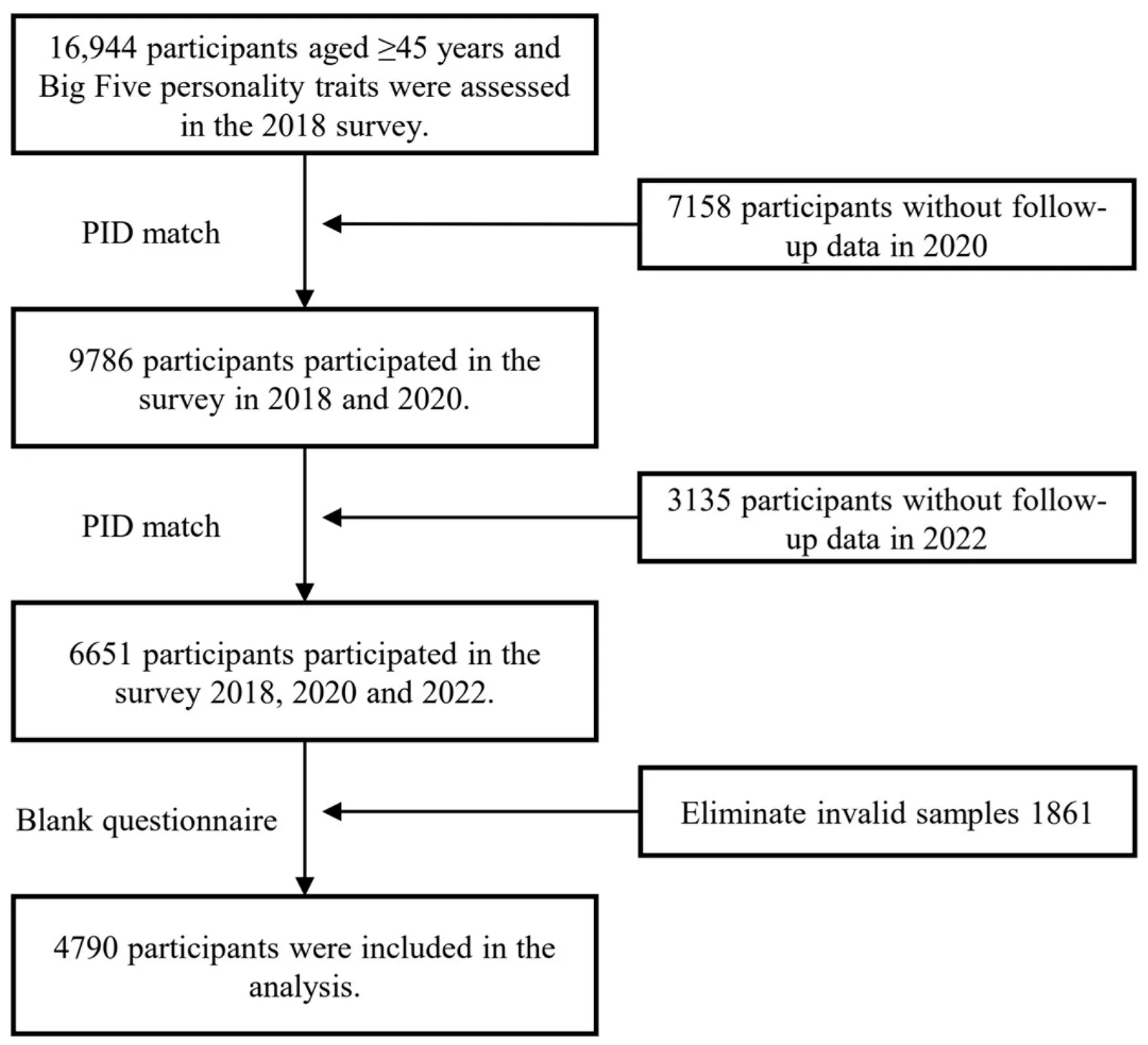
Flowchart depicting participant inclusion and exclusion.

**Figure 2:**
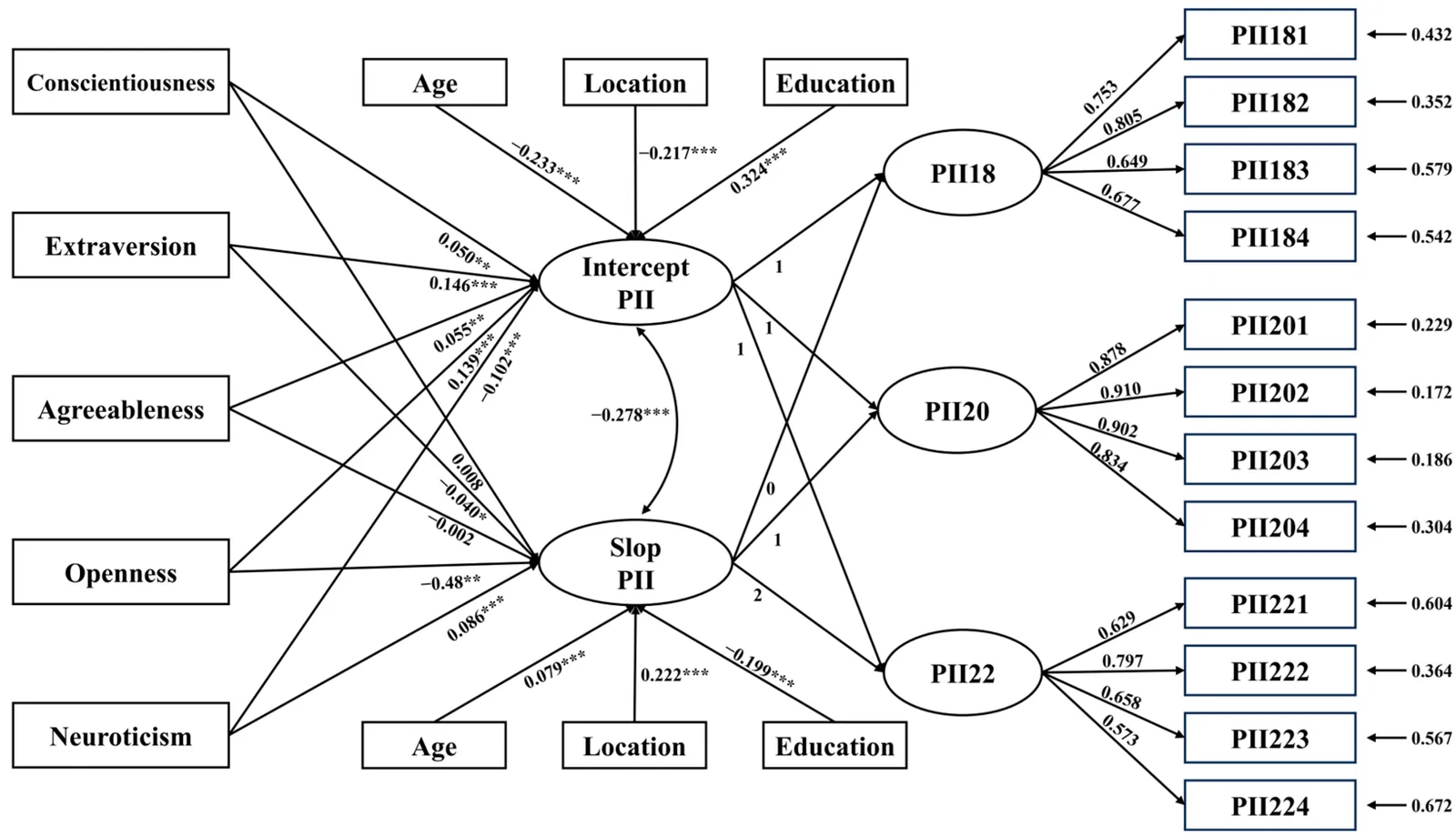
Associations of personality traits with the initial level and rate of change in PII among middle-aged and older adults. Note: * *p* < 0.05, ** *p* < 0.01, *** *p* < 0.001. PII18 represents the perceived importance of the internet in 2018, PII20 in 2020, PII22 in 2022.

**Figure 3:**
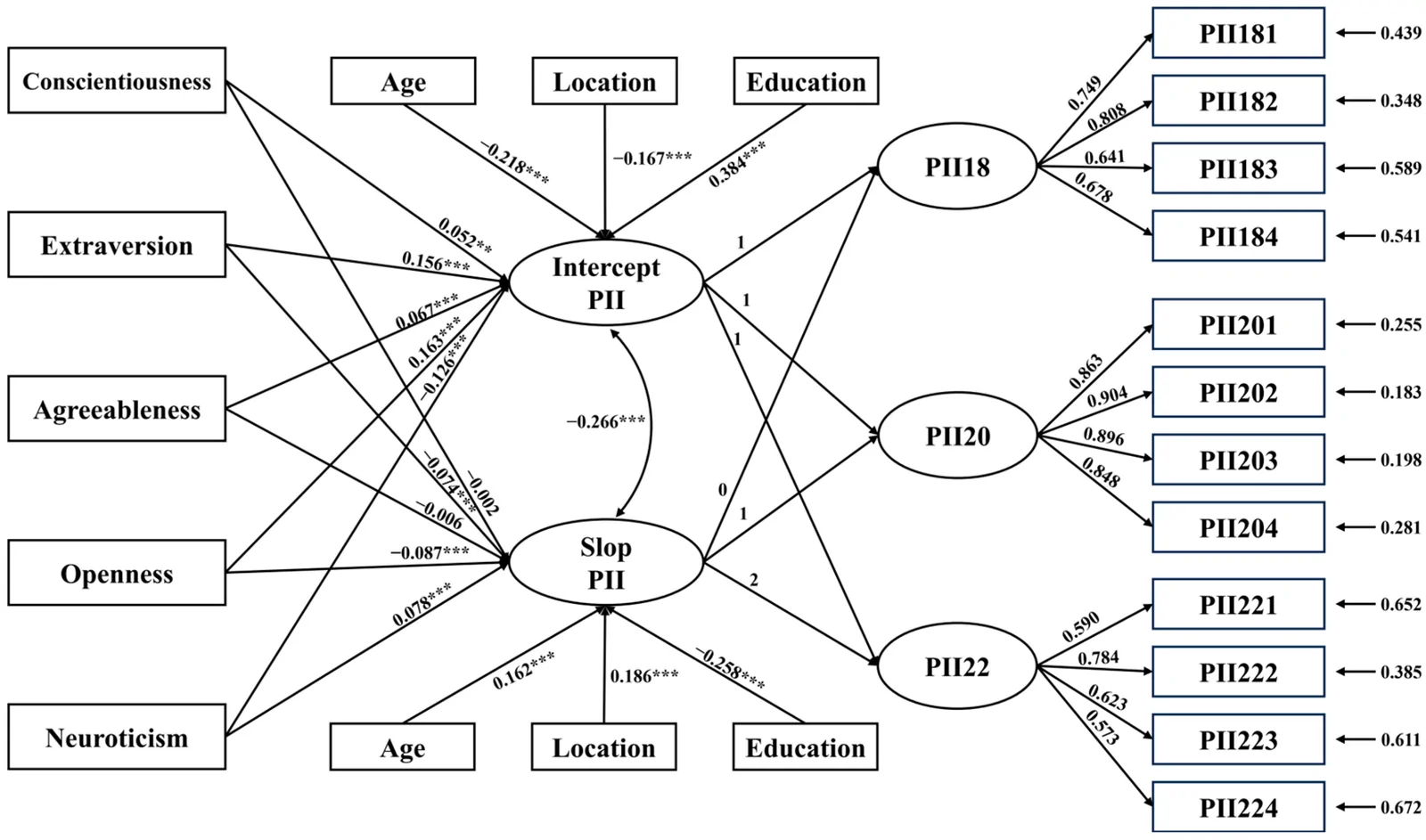
Associations of personality traits with the initial level and rate of change in PII among middle-aged adults. Note: ** *p* < 0.01, *** *p* < 0.001. PII18 represents the perceived importance of the internet in 2018, PII20 in 2020, PII22 in 2022.

**Figure 4:**
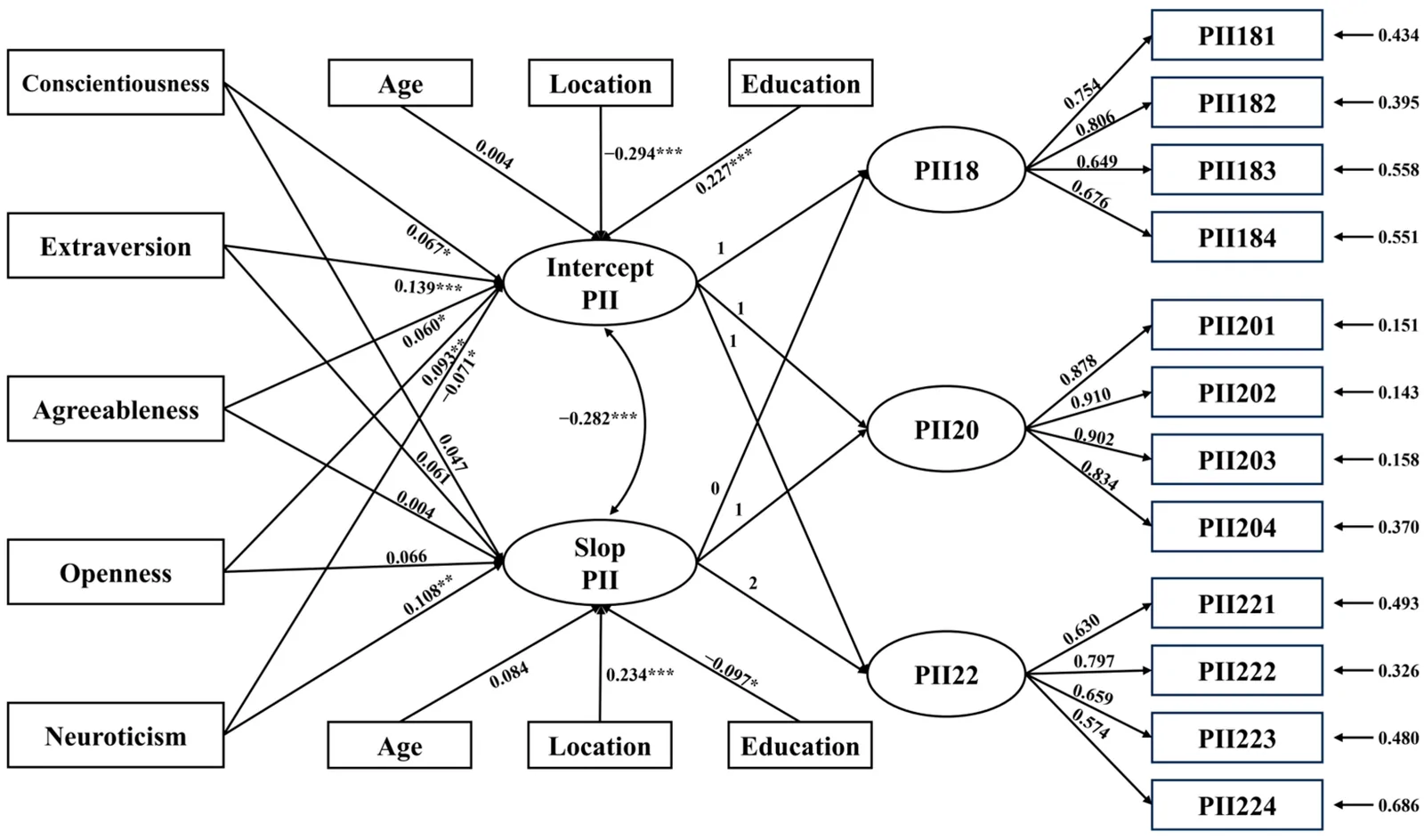
Associations of personality traits with the initial level and rate of change in PII among older adults. Note: * *p* < 0.05, ** *p* < 0.01, *** *p* < 0.001. PII18 represents the perceived importance of the internet in 2018, PII20 in 2020, PII22 in 2022.

**Table 1:** Descriptive statistics of the sample (*n* = 4790).

Demographic Variables	*n* (%)	PII18			PII20				PII22	
		Mean (SD)	*t*/*F*	*p*	Mean (SD)	*t*/*F*	*p*	Mean (SD)	*t*/*F*	*p*
Gender			1.497	0.134		2.884	0.004		2.203	0.028
Male	2502 (52.23)	7.153 (4.232)			10.685 (5.977)			14.388 (4.290)		
Female	2288 (47.77)	6.972 (4.123)			10.188 (5.930)			14.118 (4.152)		
Age group			8.442	<0.001		10.306	<0.001		11.459	<0.001
Middle-aged group	3591 (74.97)	7.360 (4.304)			10.955 (5.974)			14.658 (4.053)		
Older-adult group	1199 (25.03)	6.191 (3.649)			8.928 (5.651)			13.064 (4.503)		
Marital Status			−0.583	0.560		0.148	0.882		1.691	0.091
No partner	339 (7.08)	7.195 (4.245)			10.401 (6.006)			13.885 (4.472)		
Have a partner	4451 (92.92)	7.057 (4.176)			10.451 (5.956)			14.288 (4.206)		
Location			20.016	<0.001		14.730	<0.001		3.518	<0.001
Rural	3176 (66.30)	6.239 (3.674)			9.563 (5.958)			14.106 (4.316)		
Urban	1614 (33.70)	8.696 (4.617)			12.188 (5.568)			14.560 (4.030)		
Education			205.138	<0.001		139.422	<0.001		28.405	<0.001
Illiterate/semi-literate	846 (17.66)	5.167 (2.568)			7.517 (5.283)			13.182 (4.601)		
primary school	1155 (24.11)	6.131 (3.534)			9.313 (5.900)			14.053 (4.281)		
junior high school	1607 (33.55)	7.103 (4.153)			10.975 (5.924)			14.404 (4.186)		
high school/secondary vocational school	851 (17.77)	8.420 (4.481)			12.298 (5.540)			14.771 (3.874)		
college and above	331 (6.91)	11.541 (4.504)			14.574 (4.302)			15.710 (3.318)		

Note: PII18 represents the perceived importance of the internet in 2018, PII20 in 2020, PII22 in 2022.

**Table 2:** Correlation matrix of personality traits and PII.

	1	2	3	4	5	6	7	8
1. Conscientiousness	1							
2. Extraversion	0.237 **	1						
3. Agreeableness	0.316 **	0.140 **	1					
4. Openness	0.257 **	0.482 **	0.080 **	1				
5. Neuroticism	−0.120 **	−0.149 **	−0.133 **	−0.042 **	1			
6. PII18	0.050 **	0.150 **	0.052 **	0.143 **	−0.104 **	1		
7. PII20	0.051 **	0.082 **	0.058 **	0.101 **	−0.075 **	0.474 **	1	
8. PII22	0.068 **	0.135 **	0.053 **	0.119 **	−0.036 **	0.284 **	0.312 **	1
Mean	11.890	10.426	11.670	9.561	8.728	7.067	10.447	14.259
SD	1.899	2.366	1.770	2.605	2.283	4.180	5.959	4.226

Note: Personality traits were measured in 2018. PII18 represents the perceived importance of the internet in 2018, PII20 in 2020, PII22 in 2022. ** *p* < 0.01.

**Table 3:** Parameter estimates of the latent growth model for PII among middle-aged and older adults.

Parameters	Estimate	*SE*	*p*
Mean			
Intercept	7.708	0.036	<0.001
Slope	1.348	0.047	<0.001
Variance			
Intercept	1.186	0.047	<0.001
Slope	0.282	0.017	<0.001
Covariance			
Intercept–slope	−0.468	0.025	<0.001

**Table 4:** Parameter estimates of the conditional latent growth model for middle-aged and older adults.

Predictor Variable (2018)	Intercept Factor of PII (*β*)	*SE*	*p*	Slope Factor of PII (*β*)	*SE*	*p*
Conscientiousness	0.050	0.015	0.001	0.008	0.021	0.636
Extraversion	0.146	0.015	<0.001	−0.040	0.023	0.019
Agreeableness	0.055	0.015	<0.001	−0.002	0.021	0.914
Openness	0.139	0.015	<0.001	−0.048	0.023	0.005
Neuroticism	−0.102	0.015	<0.001	0.086	0.020	<0.001
Age	−0.233	0.015	<0.001	0.079	0.020	<0.001
Location	−0.217	0.017	<0.001	0.222	0.023	<0.001
Education	0.324	0.017	<0.001	−0.199	0.023	<0.001

**Table 5:** Parameter estimates of the conditional latent growth model for middle-aged adults.

Predictor Variable (2018)	Intercept Factor of PII (*β*)	*SE*	*p*	Slope Factor of PII (*β*)	*SE*	*p*
Conscientiousness	0.052	0.018	0.003	−0.002	0.024	0.920
Extraversion	0.156	0.020	<0.001	−0.074	0.026	<0.001
Agreeableness	0.067	0.018	<0.001	−0.006	0.023	0.777
Openness	0.163	0.020	<0.001	−0.087	0.026	<0.001
Neuroticism	−0.126	0.018	<0.001	0.078	0.023	<0.001
Age	−0.218	0.018	<0.001	0.162	0.023	<0.001
Location	−0.167	0.020	<0.001	0.186	0.025	<0.001
Education	0.384	0.021	<0.001	−0.258	0.027	<0.001

**Table 6:** Parameter estimates of the conditional latent growth model for older adults.

Predictor Variable (2018)	Intercept Factor of PII (*β*)	*SE*	*p*	Slope Factor of PII (*β*)	*SE*	*p*
Conscientiousness	0.067	0.028	0.017	0.047	0.036	0.187
Extraversion	0.139	0.027	<0.001	0.061	0.035	0.085
Agreeableness	0.060	0.028	0.031	0.004	0.036	0.899
Openness	0.093	0.028	0.001	0.066	0.036	0.065
Neuroticism	−0.071	0.028	0.012	0.108	0.036	0.003
Age	0.004	0.029	0.900	−0.084	0.044	0.063
Location	−0.294	0.034	<0.001	0.234	0.052	<0.001
Education	0.227	0.033	<0.001	−0.097	0.048	0.048

**Table 7:** Sensitivity analysis of personality traits predicting PII trajectories.

Predictor Variable (2018)	Intercept Factor of PII (*β*)	*SE*	*p*	Slope Factor of PII (*β*)	*SE*	*p*
Conscientiousness	0.006	0.016	0.699	0.026	0.021	0.225
Extraversion	0.100	0.017	<0.001	−0.020	0.023	0.374
Agreeableness	0.012	0.015	0.424	−0.029	0.021	0.167
Openness	0.104	0.017	<0.001	−0.062	0.023	0.007
Neuroticism	−0.047	0.015	0.002	0.071	0.020	0.001
Age	−0.250	0.015	<0.001	0.086	0.021	<0.001
Location	−0.222	0.017	<0.001	0.217	0.023	<0.001
Education	0.306	0.017	<0.001	−0.190	0.023	<0.001
Self-rated health	0.009	0.015	0.568	−0.049	0.021	0.018
Income status	0.005	0.015	0.761	0.029	0.020	0.147

## Data Availability

The datasets presented in this study can be found in online repositories. The names of the repository/repositories and accession number(s) can be found here: China Family Panel Studies (CFPS), https://cfpsdata.pku.edu.cn (assessed on 13 August 2026).
